# Supplementary material for: Protein language models trained on multiple sequence alignments learn phylogenetic relationships
Source: Nat Commun. 2022 Oct 22;13:6298. doi: 10.1038/s41467-022-34032-y (PMC9588007; doi:10.1038/s41467-022-34032-y)
Supplement: Supplementary file 1 — Supplementary material [file 41467_2022_34032_MOESM1_ESM.pdf]

Supplementary material for "Protein language models trained on multiple sequence alignments learn phylogenetic relationships"

Umberto Lupo[1,2,*], Damiano Sgarbossa[1,2], Anne-Florence Bitbol[1,2,*]

1 Institute of Bioengineering, School of Life Sciences, École Polytechnique Fédérale de Lausanne (EPFL), CH-1015 Lausanne, Switzerland
2 SIB Swiss Institute of Bioinformatics, CH-1015 Lausanne, Switzerland
* Corresponding authors: umberto.lupo@epfl.ch, anne-florence.bitbol@epfl.ch

Supplementary tables

		Seed MSA		Full MSA			PDB structure	
Pfam ID	Family name	L	M	L	M	$M_{\text{eff}}^{(0.2)}$	ID	Resol.
PF00004	AAA	132	207	132	39277	9050	4D81	2.40 Å
PF00005	ABC_tran	137	55	137	68891	43882	1L7V	3.20 Å
PF00041	fn3	85	98	85	42721	17783	3UP1	2.15 Å
PF00072	Response_reg	112	52	112	73063	40180	3ILH	2.59 Å
PF00076	RRM_1	68	70	69	51964	20276	3NNH	2.75 Å
PF00096	zf-C2H2	23	159	23	38996	12581	4R2A	1.59 Å
PF00153	Mito_carr	97	160	94	93776	17860	1OCK	2.20 Å
PF00271	Helicase_C	111	421	111	66809	25018	3EX7	2.30 Å
PF00397	WW	31	448	31	39045	3361	4REX	1.60 Å
PF00512	HisKA	67	265	66	154998	67303	3DGE	2.80 Å
PF00595	PDZ	82	44	82	71303	4053	1BE9	1.82 Å
PF01535	PPR	31	458	31	109064	37514	4M57	2.86 Å
PF02518	HATPase_c	112	500	111	80714	59190	3G7E	2.20 Å
PF07679	I-set	90	48	90	36141	14611	1FHG	2.00 Å
PF13354	Beta-lactamase2	215	76	198	4642	3535	6QW8	1.10 Å

Supplementary Table 1: **Pfam families and MSAs considered in this work.** For each family, we considered a shallow MSA constructed from the corresponding Pfam seed alignment (Seed MSA) and a deep MSA constructed from the Pfam full alignment (Full MSA) – see "Datasets". For both kinds of MSA, we report the length L and depth M. For the full MSAs, we report the effective depth $M_{\text{eff}}^{(0.2)}$ as defined in Equation (1). The PDB structures used, and their resolutions, are also reported. Note that occasional length mismatches between seed and full MSAs reflect our use of data from a more recent Pfam release in the case of the seed MSAs.

Family	R^2	Pearson	Slope
PF00004	0.84	0.95	0.95
PF00005	0.72	0.92	0.75
PF00041	0.56	0.90	0.75
PF00072	0.66	0.90	0.71
PF00076	0.59	0.88	0.68
PF00096	0.57	0.88	0.73
PF00153	0.81	0.93	0.80
PF00271	0.77	0.93	1.11
PF00397	0.23	0.84	1.13
PF00512	0.77	0.93	0.94
PF00595	0.50	0.89	0.63
PF01535	0.54	0.86	1.18
**PF02518**	**0.60**	**0.90**	**1.20**
**PF07679**	**0.28**	**0.85**	**0.57**
**PF13354**	**0.67**	**0.92**	**0.70**

Supplementary Table 2: **Quality of fit for our logistic model trained on Hamming distances and column attentions from several MSAs.** For the logistic model described in "MSA Transformer learns a universal representation of Hamming distances", and for the MSAs in the training set (plain font) and test set (boldface font), we report (1) the R^2 coefficient of determination for the model's predictions, (2) the Pearson correlation coefficient between predictions and ground truth Hamming distances, and (3) the slope of the line of best fit when regressing the ground truth Hamming distances on the model's predictions.

Family	R^2	Pearson	Slope
PF00004	0.31	0.67	1.60
PF00005	0.33	0.59	0.96
PF00041	0.02	0.43	1.02
PF00072	0.45	0.67	1.07
PF00076	0.30	0.56	1.10
PF00096	-0.13	0.24	0.52
PF00153	0.07	0.32	0.66
PF00271	0.13	0.46	1.25
PF00397	-0.31	0.39	1.19
PF00512	0.02	0.35	0.73
PF00595	0.49	0.70	1.09
PF01535	-0.17	0.20	0.63
**PF02518**	**-0.09**	**0.32**	**1.07**
**PF07679**	**0.29**	**0.57**	**0.91**
**PF13354**	**0.35**	**0.62**	**1.15**

Supplementary Table 3: **Quality of fit for a logistic model trained on Hamming distances and column attentions from several MSAs, using MSA Transformer with random weights.** We reinitialized MSA Transformer's parameters to random values, using the same protocols originally used in pre-training (see "MSA Transformer learns a universal representation of Hamming distances"). Results for the same task as in Supplementary Table 2 are shown.

Family	Pearson	Z-score
PF00004	0.36	-2.29
PF00005	0.46	-2.78
PF00041	0.38	-2.21
PF00072	0.41	-1.60
PF00076	0.53	-1.64
PF00096	0.88	-1.71
PF00153	0.66	-3.06
PF00271	0.83	-2.24
PF00397	0.79	-1.21
PF00512	0.66	-1.73
PF00595	0.13	0.28
PF01535	0.89	-1.68
PF02518	0.81	-2.48
PF07679	0.66	-2.20
PF13354	0.39	-2.96

Supplementary Table 4: **Relation between entropy and test error when using parameters from our common logistic model on individual column attention matrices.** We applied the common logistic model described in "MSA Transformer learns a universal representation of Hamming distances" – which was trained on the column-wise means of MSA Transformer's column attention heads [see Equation (4)] – to individual column attention heads. We then computed the resulting errors in the prediction of Hamming distances. For each of our seed MSAs, we report the Pearson correlation between the entropy of each column and the standard deviation of the distribution of these errors. We also computed the mean of this standard deviation when restricting to the 5 columns with lowest entropy. Z-scores for this mean are reported, showing that these 5 columns have significantly lower standard deviations than the rest (except for PF00595).

	PPV				Median distance (Å)			
	plmDCA		MSA Trans.		plmDCA		MSA Trans.	
Pfam ID	Eq.	Tree	Eq.	Tree	Eq.	Tree	Eq.	Tree
PF00004	0.27	0.06	0.50	0.14	8.5	13.8	5.2	9.1
PF00005	0.27	0.11	0.44	0.18	8.4	15.3	6.5	8.8
PF00041	0.33	0.16	0.45	0.35	7.3	12.1	5.2	7.0
PF00072	0.43	0.21	0.56	0.32	6.1	8.6	4.7	6.8
PF00076	0.45	0.25	0.61	0.42	6.4	9.4	4.4	6.7
PF00153	0.22	0.14	0.30	0.23	10.1	12.5	8.6	10.2
PF00271	0.30	0.14	0.50	0.16	7.7	10.5	5.1	7.8
PF00397	0.40	0.21	0.61	0.27	6.0	10.6	3.7	8.5
PF00512	0.24	0.18	0.34	0.32	9.5	11.1	7.9	7.8
PF00595	0.35	0.16	0.60	0.29	6.8	10.0	4.2	8.3
PF01535	0.37	0.23	0.45	0.34	7.5	9.2	7.1	7.6
PF02518	0.25	0.15	0.33	0.30	9.2	11.2	7.7	8.9
PF07679	0.42	0.23	0.54	0.33	4.3	8.5	3.9	6.2
PF13354	0.17	0.07	0.52	0.17	11.0	15.2	3.7	7.6
Average	0.32	0.17	0.48	0.27	7.8	11.3	5.6	7.9

Supplementary Table 5: **Comparing predicted contact maps using plmDCA and MSA Transformer on synthetic MSAs with experimental structures, for 14 of our full MSAs.** For each of the Pfam families in Supplementary Figure 4 we obtained experimental contact maps, using the PDB structures in Supplementary Table 1, by selecting the $2L$ closest residue pairs at positions i, j with $|i-j| \geq 5$. Using these structural contacts as ground truths, we computed the positive predictive values (PPVs) of the $2L$ top-scoring pairs, according to plmDCA or MSA Transformer, when performing contact inference on the synthetic MSAs generated by our Potts models either without phylogeny (Equilibrium) or with phylogeny (Tree) – see "Synthetic MSA generation via Potts model sampling along inferred phylogenies". We also report the median across predicted pairs of the inter-residue distance (all-atom minimal distance) in the reference experimental 3D structure (Median distance).

Supplementary figures

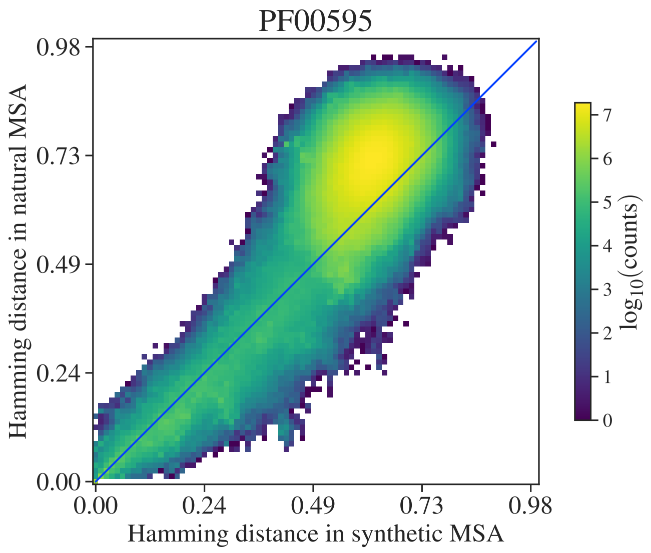

(a)

Family	Pearson correlation All	Pearson correlation $d_{\mathrm{nat}} \leq 0.5$
PF00004	0.72	0.75
PF00005	0.31	0.62
PF00041	0.31	0.90
PF00072	0.33	0.85
PF00076	0.25	0.84
PF00096	0.32	0.23
PF00153	0.39	0.91
PF00271	0.37	0.70
PF00397	0.60	0.73
PF00512	0.30	0.65
PF00595	0.67	0.96
PF01535	0.15	0.70
PF02518	0.39	0.67
PF07679	0.19	0.90
PF13354	0.74	0.79

(b)

Supplementary Figure 1: **Comparing Hamming distances from synthetic MSAs generated along inferred phylogenies with Hamming distances from natural MSAs.** The Hamming distances between sequences in our synthetic MSAs generated along phylogenies (see "Generating sequences along an inferred phylogeny under a Potts model") are reasonably correlated with those between corresponding sequences in the natural MSAs used to infer the phylogenies. (a) Density plot comparing the Hamming distances between sequences in the synthetic MSA corresponding to family PF00595, with those between corresponding sequences in the natural MSA for this family. The pixel with coordinates $(d_{\mathrm{synth}}, d_{\mathrm{nat}})$ is colored according to the base-10 logarithm of the number of pairs of indices (i, j) such that the Hamming distance between row i and row j in the synthetic MSA is d_{synth}, and the distance between the same rows in the natural MSA is d_{nat}. Recall that the sequence at row i of the synthetic MSA has been generated on the leaf of the inferred phylogenetic tree that corresponds to the natural sequence at row i of the natural MSA. As is visible here, our synthetic MSAs tend to have slightly smaller Hamming distances, on average, than their natural counterparts. (b) For each Pfam family, we computed two Pearson correlation coefficients: first, the correlation between all Hamming distances among synthetic sequences and all Hamming distances among natural sequences; second, the same correlation but restricting to pairs of indices (i, j) such that the distance between sequence i and sequence j in the natural MSA is no larger than 0.5.

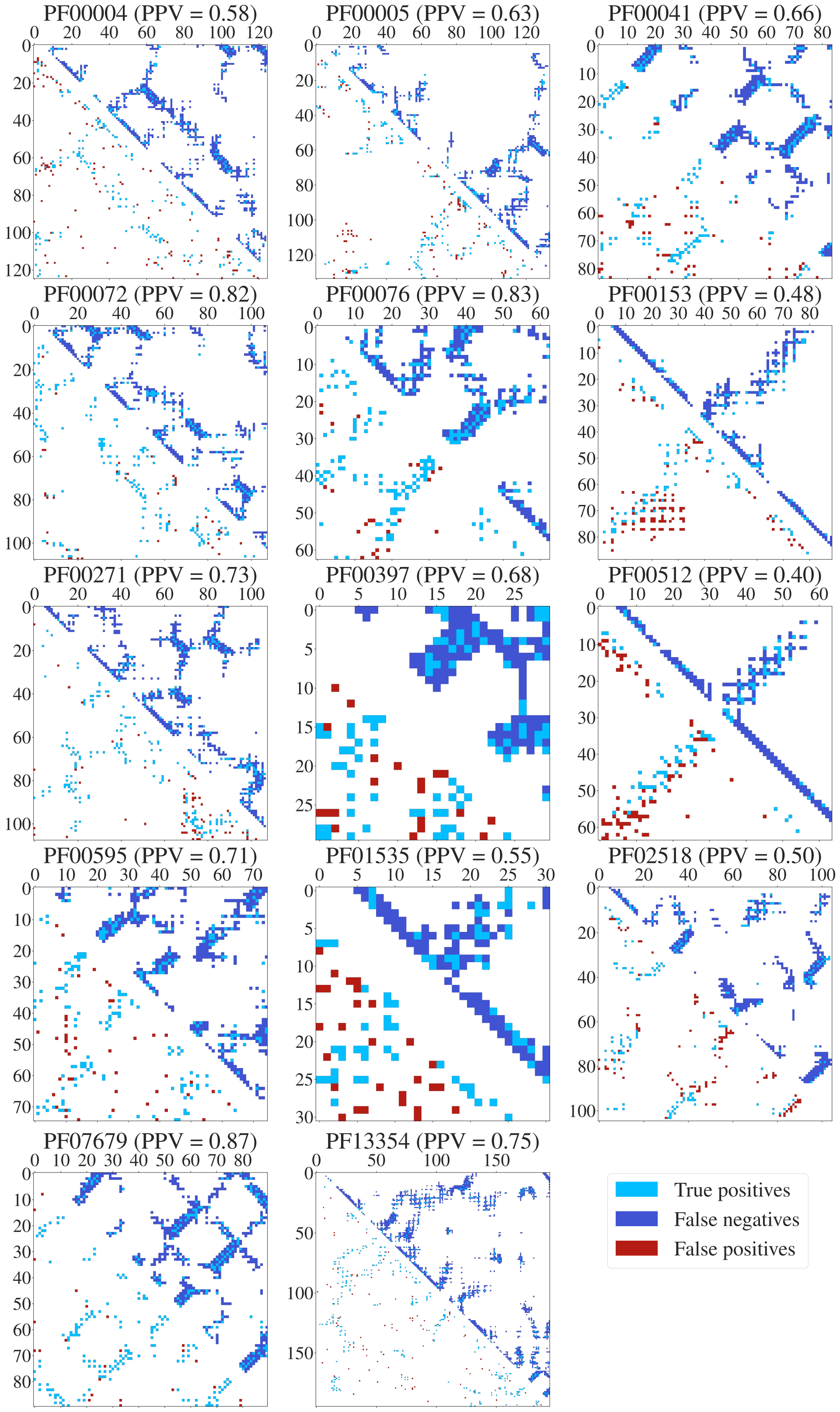

Supplementary Figure 2: **Predicted contact maps using plmDCA versus experimental contact maps for 14 of our full natural MSAs.** Experimental contact maps are displayed in the upper-triangular portions of each panel, and are obtained from the PDB structures in Supplementary Table 1 by using an all-atom minimal Euclidean distance cutoff of 8 Å, excluding residue pairs at positions i, j with $|i - j| \leq 4$. Predictions are displayed in the lower-triangular portions, and are obtained considering the top $2L$ scores, where L is the length of the MSA. Light blue squares represent true positive predictions, dark blue squares false negative predictions, and red squares false positive predictions. For each predicted contact map, we report the positive predictive value (PPV) given these choices. Results for PF00096 are not displayed due to its very short length.

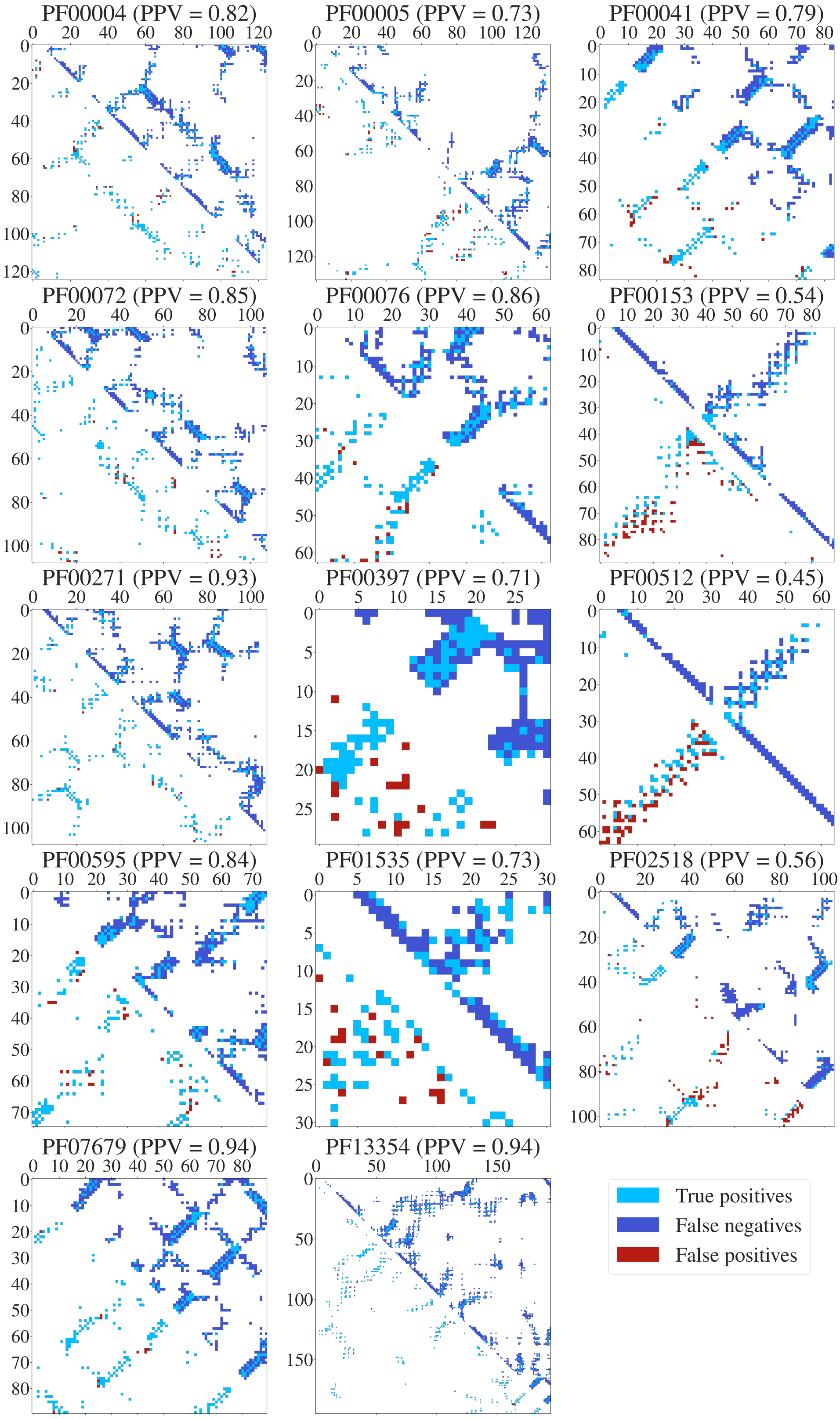

Supplementary Figure 3: **Predicted contact maps using MSA Transformer versus experimental contact maps for 14 of our full natural MSAs.** Same as in Supplementary Figure 2, but using MSA Transformer instead of plmDCA to infer structural contacts. Contact scores were computed for each natural MSA as follows: (1) the MSA was subsampled 10 times using the same randomly sampled row indices used for the corresponding synthetic MSAs (see "Assessing performance degradation due to phylogeny in coupling inference"); (2) for each subsample, a matrix of contact scores was computed using MSA Transformer's row attention heads and the estimated contact probabilities from the logistic regression trained in [28]; (3) the resulting 10 matrices were averaged to obtain a single matrix of contact scores.

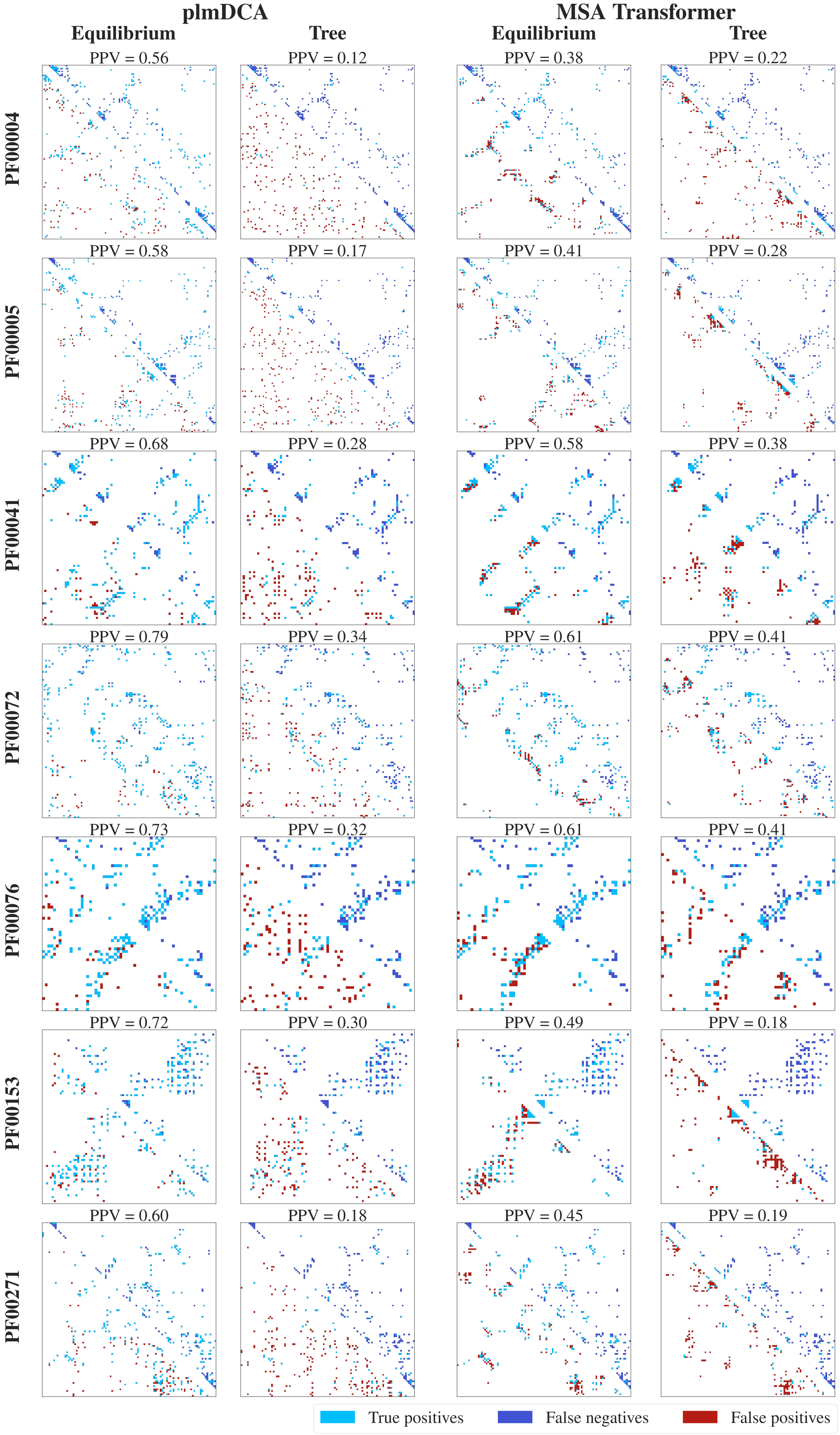

Supplementary Figure 4: (Continued on the next page.)

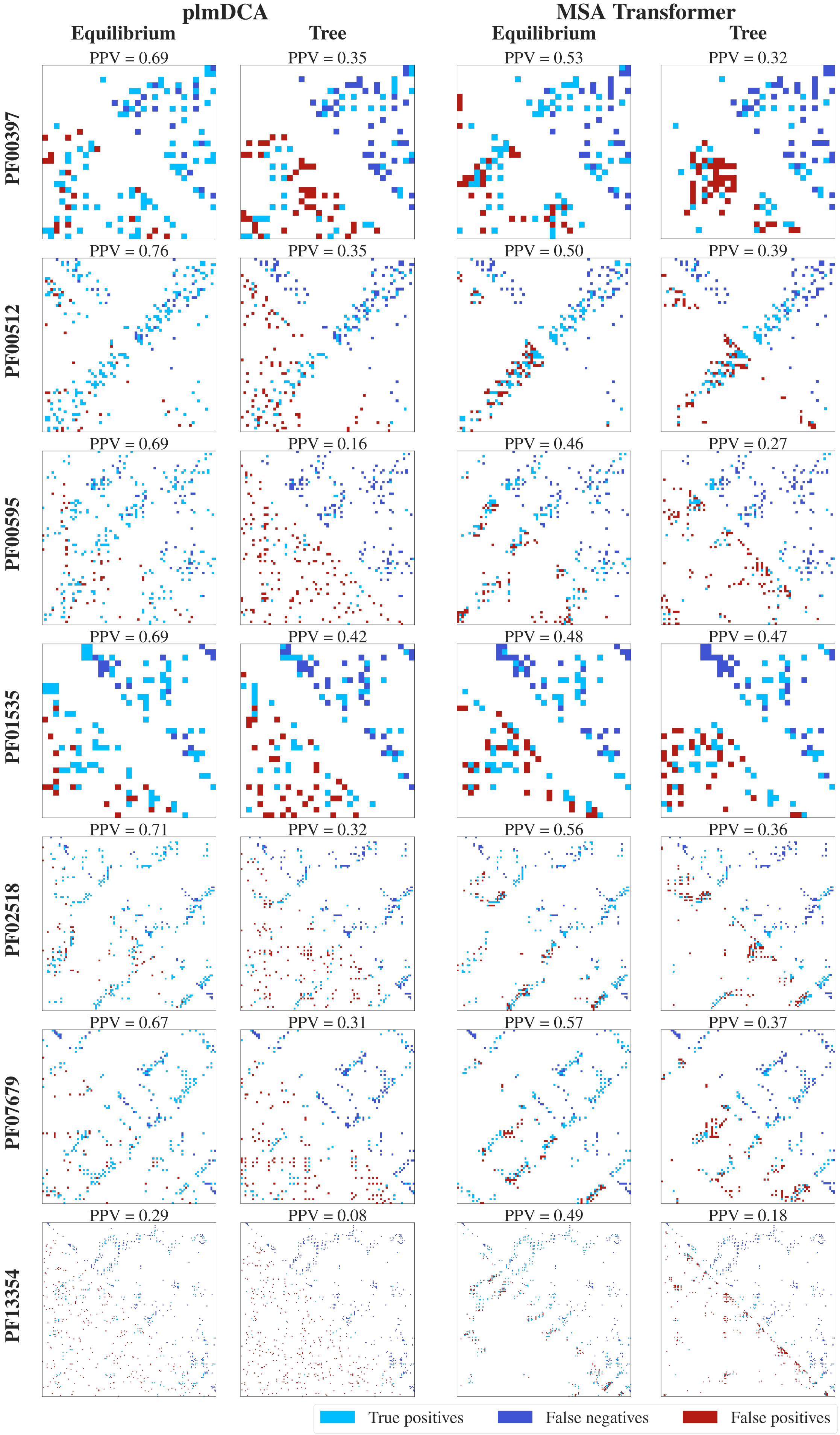

Supplementary Figure 4: (Continued on the next page.)

Supplementary Figure 4: **Predicted contact maps using plmDCA and MSA Transformer on synthetic MSAs, versus ground truth proxy contact maps defined by top Potts model couplings, for 14 of our full MSAs.** Contact maps containing $2L$ contacts, and obtained from the ground-truth couplings in the Potts models used to generate our synthetic MSAs (see "Synthetic MSA generation via Potts model sampling along inferred phylogenies"), are displayed in the upper-triangular portions of each panel. In the lower-triangular portions, we display the $2L$ top-scoring pairs according to plmDCA or MSA Transformer, when performing contact inference on synthetic MSAs generated from those Potts models either without phylogeny (Equilibrium) or with phylogeny (Tree). Light blue squares represent true positive predictions, dark blue squares false negative predictions, and red squares false positive predictions. For each predicted contact map, we report the positive predictive value (PPV) given these choices. ROC-AUC values corresponding to this analysis are reported in the columns titled "ROC-AUC for $2L$ contacts" of Table 2.
