# Supplementary material for: Protein language models trained on multiple sequence alignments learn phylogenetic relationships
Source: Nat Commun. 2022 Oct 22;13:6298. doi: 10.1038/s41467-022-34032-y (PMC9588007; doi:10.1038/s41467-022-34032-y)
Supplement: Supplementary file 2 — Reporting Summary [file 41467_2022_34032_MOESM2_ESM.pdf]

nature portfolio

Corresponding author(s): Umberto Lupo
Anne-Florence Bitbol

Last updated by author(s): Sep 20, 2022

Reporting Summary

Nature Portfolio wishes to improve the reproducibility of the work that we publish. This form provides structure for consistency and transparency in reporting. For further information on Nature Portfolio policies, see our Editorial Policies and the Editorial Policy Checklist.

Statistics

For all statistical analyses, confirm that the following items are present in the figure legend, table legend, main text, or Methods section.

n/a	Confirmed	
☐	☒	The exact sample size (*n*) for each experimental group/condition, given as a discrete number and unit of measurement
☐	☒	A statement on whether measurements were taken from distinct samples or whether the same sample was measured repeatedly
☒	☐	The statistical test(s) used AND whether they are one- or two-sided<br>*Only common tests should be described solely by name; describe more complex techniques in the Methods section.*
☐	☒	A description of all covariates tested
☒	☐	A description of any assumptions or corrections, such as tests of normality and adjustment for multiple comparisons
☐	☒	A full description of the statistical parameters including central tendency (e.g. means) or other basic estimates (e.g. regression coefficient) AND variation (e.g. standard deviation) or associated estimates of uncertainty (e.g. confidence intervals)
☒	☐	For null hypothesis testing, the test statistic (e.g. *F*, *t*, *r*) with confidence intervals, effect sizes, degrees of freedom and *P* value noted<br>*Give P values as exact values whenever suitable.*
☒	☐	For Bayesian analysis, information on the choice of priors and Markov chain Monte Carlo settings
☒	☐	For hierarchical and complex designs, identification of the appropriate level for tests and full reporting of outcomes
☐	☒	Estimates of effect sizes (e.g. Cohen's *d*, Pearson's *r*), indicating how they were calculated

Our web collection on statistics for biologists contains articles on many of the points above.

Software and code

Policy information about availability of computer code

Data collection	Raw data was collected from public sources as specified in the "Data" section below. No specific software was used to this end.
Data analysis	No commercial software was used to perform data analysis. The programming languages used were Python (version 3.9), Julia (version 1.7) and C++ (GCC compiler version 8.4.0).<br>We used the HMMER suite (http://hmmer.org, version 3.3.2) to align the Pfam seed alignments to their HMMs.<br>We used the fair-esm Python library (version 0.4.0) to run the pre-trained MSA Transformer model on our MSAs.<br>We used the statsmodel Python library (version 0.13.2) to train logistic models on the column attention matrices computed by MSA Transformer.<br>We used the bmDCA software (https://github.com/ranganathanlab/bmDCA, version 0.8.12) to infer Potts model Hamiltonians from our deep multiple sequence alignments (MSAs), and to generate synthetic MSAs at equilibrium.<br>We used FastTree (http://www.microbesonline.org/fasttree/, version 2.1) to infer unrooted phylogenetic trees from our deep MSAs.<br>We used open-source code available at https://github.com/Bitbol-Lab/Phylogeny-Partners (version 2.0) to generate synthetic MSAs along a phylogenetic tree using the Metropolis criterion and the inferred bmDCA Potts model Hamiltonians.<br>We used the PlmDCA Julia package (https://github.com/pagnani/PlmDCA, version 0.4.1) to infer contact maps from our natural and synthetic MSAs.<br><br>Additional custom code, and instructions for reproducing our analysis, are available in the Zenodo archive https://zenodo.org/record/7096792.

For manuscripts utilizing custom algorithms or software that are central to the research but not yet described in published literature, software must be made available to editors and reviewers. We strongly encourage code deposition in a community repository (e.g. GitHub). See the Nature Portfolio guidelines for submitting code & software for further information.

Data

Policy information about availability of data

All manuscripts must include a data availability statement. This statement should provide the following information, where applicable:

- Accession codes, unique identifiers, or web links for publicly available datasets
- A description of any restrictions on data availability
- For clinical datasets or third party data, please ensure that the statement adheres to our policy

Raw data was collected from three public sources: 1) natural multiple sequence alignments (MSAs) from the bmDCA open-source repository (https://github.com/matteofigliuzzi/bmDCA); 2) natural MSAs and hidden Markov models (HMMs) from the Pfam database (https://pfam.xfam.org/, version 35.0); 3) protein structures from the Protein Data Bank (PDB) (https://www.rcsb.org/).

The full list of natural MSAs and PDB structures, described in the "Datasets" subsection of the main text, is in Supplementary Table 1, in which PDB accession numbers and Pfam IDs are provided.

All datasets used in our analysis are contained in the Zenodo archive https://zenodo.org/record/7096792.

Field-specific reporting

Please select the one below that is the best fit for your research. If you are not sure, read the appropriate sections before making your selection.

☐ Life sciences ☐ Behavioural & social sciences ☒ Ecological, evolutionary & environmental sciences

For a reference copy of the document with all sections, see nature.com/documents/nr-reporting-summary-flat.pdf

Ecological, evolutionary & environmental sciences study design

All studies must disclose on these points even when the disclosure is negative.

Study description

Our work was purely computational and did not involve any experiments.

We performed two computational studies. In the first, we demonstrated that the deep learning model "MSA Transformer", when given a multiple sequence alignment (MSA) as input, encodes the Hamming distances between sequences in the MSA as a simple combination of its column attention matrices. In the second study, we provided evidence that MSA Transformer decouples phylogenetic signal from functional constraints, by generating synthetic MSAs modelled on natural MSAs, first without phylogeny and then along a phylogeny, and showing that MSA Transformer's contact inference is more robust to the addition of phylogeny than contact inference by direct coupling analysis (DCA) models.

For both these studies, we considered 15 different Pfam families, listed in Supplementary Table 1.

Research sample

Natural MSAs were collected from the bmDCA open-source repository (https://github.com/matteofigliuzzi/bmDCA) and from the Pfam database (https://pfam.xfam.org/, version 35.0). Protein structures were collected from the Protein Data Bank (PDB) (https://www.rcsb.org/). The full list of natural MSAs and PDB structures, described in the "Datasets" subsection of the main text, is in Supplementary Table 1, in which PDB accession numbers and Pfam IDs are provided.

The choice of Pfam seed MSAs for our first study is motivated by the fact that these are curated MSAs with sufficient diversity and appropriate size for MSA Transformer. For our second study, the use of some deep MSAs previously published in https://github.com/matteofigliuzzi/bmDCA makes our results readily comparable with the literature.

While the seed MSAs represent a well-studied and small portion of the entire ensemble of protein homologs belonging to a Pfam family, the full MSAs include many more homologs, including some distant ones but also some homologs that are quite close in sequence.

Sampling strategy

Sample sizes were adequate to our two studies. In our study on how MSA Transformer captures phylogenetic relationships in natural data, we first performed several logistic-type regressions to fit 145 parameters, using between thousands and hundreds of thousands of samples in each case. The main result in this first study involved fitting 145 parameters using several million samples. In our second study, we ensured that the depths of the natural MSAs used were large enough for the DCA models used (bmDCA, plmDCA) to fit properly.

Our analysis on the impact of phylogeny on the inference of couplings from synthetic MSAs involved random subsamplings of the generated MSAs. This was necessary so that the computations made by MSA Transformer could fit in the available RAM. Our results were robust to the use of different random subsamplings.

Data collection

One of the authors (UL) collected data from the online sources listed in "Data".

Timing and spatial scale

The Pfam seed MSAs used in our analysis on MSA Transformer's column attention were fetched from the Pfam website on 22 December 2021. Some of the Pfam full MSAs used in our analysis of contact inference come from the bmDCA repository (https://github.com/matteofigliuzzi/bmDCA), where they were added on 11 December 2017, while we fetched the remaining Pfam full MSAs

	ourselves on 20 April 2021. This temporal gap is not the source of biases in our analysis, as the hidden Markov models used by Pfam have changed very little in recent years. Instead, the use of some MSAs already analysed in the literature allows for comparison with previously published results.
Data exclusions	In the case of family PF02518, out of the initial 658 sequences from the Pfam seed MSA, we kept only the first 500 in order to limit the memory requirements of our computational experiments to less than 64GB. This criterion was pre-determined and we never considered the remaining 158 sequences.
Reproducibility	Our analysis on the impact of phylogeny on the inference of couplings from synthetic MSAs involved random subsamplings of the generated MSAs. We repeated this part of the analysis by using two additional sets of new random splittings, namely: i) one new set of random splittings with the same depths as in our final analysis; ii) one new set of random splittings using twice-deeper subsampled MSAs. We thus found that our conclusions in this section were reproducible.<br><br>Our study on how MSA Transformer captures phylogenetic relationships in natural data was deterministic and hence not affected by reproducibility concerns.
Randomization	Our computational analyses involve random splittings into training and test sets, random subsamplings, and/or independent random initializations of our sampling algorithms.
Blinding	Blinding was not relevant to our study as it is a purely computational experiment and as the raw data used was from publicly available sources with next to no data exclusions.

Did the study involve field work? ☐ Yes ☒ No

Reporting for specific materials, systems and methods

We require information from authors about some types of materials, experimental systems and methods used in many studies. Here, indicate whether each material, system or method listed is relevant to your study. If you are not sure if a list item applies to your research, read the appropriate section before selecting a response.

Materials & experimental systems

n/a	Involved in the study
☒	☐ Antibodies
☒	☐ Eukaryotic cell lines
☒	☐ Palaeontology and archaeology
☒	☐ Animals and other organisms
☒	☐ Human research participants
☒	☐ Clinical data
☒	☐ Dual use research of concern

Methods

n/a	Involved in the study
☒	☐ ChIP-seq
☒	☐ Flow cytometry
☒	☐ MRI-based neuroimaging
